# Nurses' stressors and their quality of life: A study on nurses caring for older patients

**DOI:** 10.1002/nop2.553

**Published:** 2020-07-06

**Authors:** Huda A. Anshasi, Mirna Fawaz, Sura Alhalalmeh, Wafa Qasem Ahmad, Ahmad Tassi

**Affiliations:** ^1^ School of Nursing The University of Jordan Amman Jordan; ^2^ Nursing Department Faculty of Health Sciences Beirut Arab University Beirut Lebanon; ^3^ Nursing Department Fatima College of Health Sciences Al Ain United Arab Emirates; ^4^ Islamic Hospital Amman Jordan

**Keywords:** care for older people, nurses, nurses' quality of life, nursing, occupational stress

## Abstract

**Aim:**

To determine the sources of occupational stress and the level of quality of life among nurses caring for older people in Lebanon and examine the underlying factors to predict nurses' quality of life.

**Design:**

A descriptive correlational design.

**Methods:**

Data were collected from 119 nurses using Nursing Stress Scale and WHO‐Quality of Life Brief.

**Results:**

Nurses reported the highest frequency of stressful events related to their workload (mean = 16.42, *SD* 1.03), followed by “death and dying” (mean = 14.61, *SD* 1.02). Nurses reported the highest level of quality of life domains was physical health (mean = 15.74, *SD* = 2.63), while the lowest level was environmental domain (mean = 11.15, *SD* = 1.86). After controlling for demographic and work‐related variables, occupational stress explained a large variance in the physical (*R* ^2^ change = .43), psychological (*R* ^2^ change = .44) and social relationship (*R* ^2^ change = .35) domains of quality of life.

## 1 INTRODUCTION AND BACKGROUND

Globally, the population aged 60 years and above is growing faster than any younger age group. According to the UN, (2017), between 2015–2050, the proportion of the world's older adults is expected to nearly double from 12%–22% (UN, 2017), whereas in Arab region, it is estimated to almost double and a half from 6.02%–15.2% (UNFPA, 2017). Lebanon has the fastest growing older adult population in the Arab region (Abdulrahim, Ajrouch, & Antonucci, 2015). By the end of 2015, people aged 60 years and older represented 7.3% of Lebanon's population and is expected to increase to 12.0% and 21.0% by the year 2030 and 2050, respectively (Sibai, Rizk, & Kronfol, 2015).

Moreover, older people are more vulnerable to chronic diseases and need more healthcare services than younger age group. The National Institute on Aging (2017) reported that 85% of older people have at least one long‐term illness and 60% have at least two conditions. Due to escalating population size and number of comorbidities among older people, it is an essential need to enhance the quality of health care among this population.

Quality of health care is defined as “providing effective and efficient healthcare services that meet both healthcare provides' and patients' satisfaction” (Mosadeghrad, 2013). Previous studies have shown that occupational stress among nurses decreases their efficiency of job performance that has a negative effect on the quality of patient care and patient satisfaction (Olayinka, Osamudiamen, & OjoAdeleke, 2013; Sarafis, 2016; Teng, Hsiao, & Chou, 2010).

Occupational stress, sometimes called job stress, is the physical and emotional reactions that happen when the requirements of the work do not match the nurse's abilities and resources (Nakhli, 2013). Nurses face extraordinary stressors in the medical environment (Alenezi, Aboshaiqah, & Baker, 2018; Alkhawaldeh et al., 2019; Alkhawaldeh et al., 2019; Galdikien, Asikainen, Balciunas, & Suominen, 2014). This is especially for older people care settings, where patients often exhibit many of symptoms such as agitation that can be stressful for the healthcare providers (Zwijsen et al., 2014).

Nurses working with older patients may experience stress more than other nurses working in different settings because of high workloads, physical and psychological strain (Elovainio et al., 2015). Physical strain may develop as a result of moving patients, assisting them in bathing, etc., whereas psychological strain may result from the inability to complete tasks or incompetency in ethical decision‐making (Bollig, Schmidt, Rosland, & Andreas Heller, 2015; Zwijsen et al., 2014).

Currently, there has been growing interest in examining occupational stress among nurses working with older patients (El‐Hneiti, Shaheen, Bani Salameh, Al‐Hussami, & Ahmad, 2019). From previous studies, two factors “workloads” and “patient‐related difficulties” were identified as most common stressors among nurses working with older patients (El‐Hneiti et al., 2019; Zwijsen et al., 2014). Exposure to a long period of these stressors can easily cause many negative effects on the nurses' physical and psychological health, including irritability, anxiety and fatigue (Engström, Ljunggren, Lindqvist, & Carlsson, 2006; Jarrad, Hammad, Shawashi, & Mahmoud, 2018). Physical and psychological functioning is directly related to perception of quality of life. Quality of life refers to multidimensional concept that includes aspects of physical and psychological health (Skevington, Lotfy, & Connell, 2004).

To date, few studies have examined the quality of life of nurses and the impact of demographic and work‐related variables and occupational stress on their health (Hamaideh, 2012; Itzhaki, 2018; Sarafis, 2016). These studies have focused on quality of life among psychiatric nurses (Hamaideh, 2012; Itzhaki, 2018) and general nurses (Sarafis et al., 2016). However, to the best of our knowledge, no study has been conducted with a focus on quality of life among nurses working with older people. Furthermore, in Lebanese, the sources of occupational stress among nurses especially nurses working with older patients have not been explored yet. Exploring the sources of occupational stressors that have an impact on nurses caring for older patients is necessary because that helps in developing appropriate interventions to reduce their stress. Therefore, the purposes of this study were as follows: (1) to determine the sources of occupational stress and the level of quality of life among nurses caring for older patients in Lebanon and (2) to examine the underlying factors to predict nurses' quality of life.

## 2 METHODS

### 2.1 Design and sample

A descriptive correlational design was used to implement the study. A convenience sample of nurses working in older adult ward was recruited from Dar AlAjaza AlIslamia hospital in Beirut, Lebanon. The operating expenses of Dar AlAjaza AlIslamia hospital are funded partially by the government and by private donations (from institutional and individual donors). Dar AlAjaza AlIslamia hospital comprises today 600 beds operated by 300 physicians (Psychiatrists, Geriatricians and other specialties) and around 350 nurses and other workers.

Registered nurses (University Education Graduates) and licensed practical nurses (a 2‐year College program in nursing) were only included in this study if they had at least 6 months of work experience in older people care settings and were willing to participate in this study.

The sample size was calculated using statistical software package G* Power 3.0.10. Based on a medium effect size, α = 0.05 and a power of 0.80, the minimum required sample was 109 participants. To be conservative and avoid the negative impact of attrition, the sample was increased to 130.

A sample of 130 participants was recruited to determine the sources of occupational stress and the level of quality of life among Lebanese nurses and of those who recruited, 119 participants completed the questionnaires (representing a response rate of 90.8%).

### 2.2 Measurement instruments

The instrument had three parts. Part A comprised the items that were used to collect the nurses' demographic and work‐related characteristics (age, gender, marital status, years of clinical experience in older people care settings, level of education, monthly salary and shift worked).

Part B was the Nursing Stress Scale (NSS). The NNS was used to determine sources of occupational stressors among nurses. It was developed based on 34 potentially stressful situations by Gray‐Toft and Anderson in 1981. They identified 7 major sources of occupational stress; one factor relates to the physical environment (workload), four factors arise from the psychological environment (death and dying), inadequate preparation to deal with the emotional needs of patients and their families, lack of support and uncertainty concerning treatment and two factors relate to the social environment (conflict with physicians) and conflict with other nurses and supervisors (Gray‐Toft & Anderson, 1981).

The NSS is scored on a 4‐point Likert scale with ranges from 0 (never)–3 (very frequently). Scoring is conducted by adding up the individual item responses for each subscale. This gives a score for each subscale. To get a total score, all 34 item responses are added together. A total score ranges from 0–102, with high scores indicating more frequent of a specific source of stress. The NSS was reported to have the internal consistency reliability ranging from 0.90–0.92 for the whole scale (Gray‐Toft & Anderson, 1981). In this study, Cronbach's alpha reliability of the NSS was 0.91.

Part C was World Health Organization‐Quality of Life Brief Questionnaire (WHOQOL‐BREF). The WHOQOL‐BREF consists of four dimensions: physical, psychological, social and environmental (Skevington et al., 2004). All items are rated on a 5‐point Likert scale with ranges from 1 (strongly agree)–5 (strongly disagree), with the highest scores representing better quality of life.

The Cronbach's alpha for the physical, psychological, social relationship and environment domains were 0.82, 0.81, 0.68 and 0.80, respectively (Skevington et al., 2004). In present study, the Cronbach's alpha coefficient of the WHOQOL‐BREF was 0.93 for all 26 questions, whereas the Cronbach's alpha coefficient for the physical, psychological, social relationship and environment domains was 0.81, 0.81, 0.69 and 0.78, respectively.

The pilot testing was conducted using 10 Lebanese nurses to evaluate the comprehensiveness and appropriateness of the study instruments; no modifications were required. The English version of two instruments was used because English is the official language for nursing education in Lebanon. Also, the sample was limited to nurses only, who could communicate in English.

### 2.3 Data collection and ethical consideration

The data were collected between January 2019–March 2019. The Institutional Review Board (IRB) of Dar AlAjaza AlIslamia hospital and Beirut Arab university approved the study protocol. Eligible participants were asked to sign informed consent after they were informed of the necessary information about the purposes, significance and risks of the study. Moreover, they were informed that anytime during the study they could withdraw without penalty. All participants were assured of confidentiality and voluntary participation. After obtaining written consent, the participants answered the questionnaires by self‐reporting.

### 2.4 Data analysis

Data were entered and processed using the Statistical Package for Social Sciences (SPSS) version 22.0. Descriptive statistics were used to describe the sample and study variables. Independent *t* test and ANOVA were conducted to assess differences in nurses' stressors and their quality of life according to demographic and work‐related variables.

Pearson product‐moment coefficients were conducted to establish associations between study variables and to identify which variables would enter regression model. Hierarchical regression analysis was conducted to identify the relative and overall contribution of demographic, work‐related variables and occupational stress on nurses' quality of life. Before regression analyses were conducted, multicollinearity was assessed. The intercorrelations between the variables were small to medium, as all tolerance statistics were more than 0.2 and the values of variance inflation factors were less than 10 for all variables.

## 3 RESULTS

### 3.1 Participants' characteristics

The study sample consisted of 119 nurses who met the inclusion criteria, of whom 59.7% were female. Most of participants were married (*N* = 87, 73.1%), holding a Baccalaureate degree (*N* = 84, 70.6%) and working rotated shift (day and night) (*N* = 71, 59.7%). Table 1 provides a summary of participant demographic and work‐related characteristics.

**Table 1:** Sample Characteristics (*N* = 119)

Variables	*N* (%)	Mean (*SD*)	Range
Age (years)		39.7 (8.2)	23–56
Gender			
Male	48 (40.3)		
Female	71 (59.7)		
Marital status			
Single	30 (25.2)		
Married	87 (73.1)		
Divorced	2 (1.7)		
Educational level			
Diploma	35 (29.4)		
Baccalaureate	84 (70.6)		
Monthly income (LBP)^a^			
800.000–999.000	35 (29.4)		
1.000.000–1.200.000	71 (59.7)		
>1.200.000	13 (10.9)		
Shift worked			
Day (12 hr)	48 (40.3)		
Rotated (day & night)	71 (59.7)		
Years of experience in older people care settings		5 (1.0)	2–15

^a^ LBP1 = US$0.0007.

### 3.2 Mean values of the NSS and WHOQOL‐BREF scales

The nurses' sum stressors mean was 77.95 (*SD* 2.03) on the NSS scale. The workload subscale was the highest (mean = 16.42, *SD* 1.03), followed by “death and dying” (mean = 14.61, *SD* 1.02), whereas the lowest subscale was “inadequate preparation” (mean = 6.33, *SD* 1.40). In regard to WHOQOL‐BREF, the highest level of quality of life domains was physical (mean = 15.74, *SD* 2.63), followed by psychological (mean = 15.52, *SD* 2.59) and social relationships (mean = 14.10, *SD* 2.35), while the lowest level of quality of life was environmental domain (mean = 11.15, *SD* 1.86).

### 3.3 Factors associated with quality of life and occupational stress

An independent *t* test and ANOVA were conducted to assess the statistically significant differences in quality of life domains according to participants' characteristics (Table 2). The results of analysis showed that females had a significant lower quality of life for physical and social relationship domains compared with males (*t* = 18.66, *p* < .01) and (*t* = 17.31, *p* < .01), respectively. Furthermore, diploma nurses had a significant lower quality of life for psychological domain compared with Baccalaureate degree nurses, at (mean = 13.67, *SD* 2.50) versus (mean = 18.13, *SD* 5.95), (*t* = 24.21, *p* < .01).

**Table 2:** Differences in occupational stress and quality of life according to demographic and related‐work variables

Variable	NSS	WHQOL‐BREF			
	Occupational stress	Physical domain	Psychological domain	Social relationships domain	Environmental domain
	Mean; *t*; *p*‐Value; (*SD*)	Mean; *t*; *p*‐Value; (*SD*)	Mean; *t*; *p*‐Value; (*SD*)	Mean; *t*; *p*‐Value; (*SD*)	Mean; *t*; *p*‐Value; (*SD*)
Gender					
Male	72.97; 0.14; .79; (7.16)	16.97; 18.66; <.01; (7.16)	14.54; 3.24; .26; (5.97)	15.95; 17.31; <.01; (7.17)	12.45; 0.20; .60; (6.07)
Female	73.32 (5.48)	13.32 (5.48)	15.32 (6.04)	12.30 (5.64)	11.02 (4.74)
Education					
Diploma	65.67; 18.66; <.01; (5.50)	14.67; 0.14; .69; (5.50)	13.67; 24.21; <.01; (2.50)	13.67; 0.15; .79; (5.49)	12.60; 0.18; .71; (7.01)
Baccalaureate	71.13 (4.95)	13.13 (4.95)	18.13 (5.95)	12.13 (4.94)	11.15 (4.95)
Shift worked					
Morning	66.67; 24.21; <.01; (7.07)	16.67; 10.63; .08; (7.07)	16.80; 3.69; .19; (7.12)	17.14; 30.14; <.01; (5.94)	12.94; 1.90; .43; (5.94)
Evening/rotating	73.98 (3.98)	14.98 (3.98)	15.21 (4.90)	13.65 (2.90)	11.25 (2.90)

	Mean; *F*; *p*‐Value; (*SD*)	Mean; *F*; *p*‐Value; (*SD*)	Mean; F; *p*‐Value; (*SD*)	Mean; *F*; *p*‐Value; (*SD*)	Mean; *F*; *p*‐Value; (*SD*)
Marital status					
Single	69.37; 0.47; .39; (5.61)	13.37; 0.47; .35; (5.61)	13.38; 0.42; .46; (5.51)	12.38; 0.46; .40; (5.50)	11.28; 0.36; .50; (5.01)
Married	70.78 (6.40)	14.78 (6.40)	14.80 (7.40)	13.80 (6.39)	12.70 (6.40)
Divorced	68.93 (5.29)	13.20 (5.29)	13.21 (5.32)	12.21 (5.32)	11.11 (4.82)
Monthly income					
(LBP)^a^					
800.000–999.000	72.38; 0.43; .44; (5.48)	13.38; 0.43; .40; (5.48)	13.37; 0.32; .54; (5.98)	12.37; 0.31; .49 (4.97)	11.37; 0.33; .53; (5.98)
100.000–1.200.000	71.21 (6.90)	13.21 (5.30)	13.20 (5.66)	12.10 (4.65)	11.20 (5.66)
>1.200.000	71.80 (7.43)	14.80 (7.43)	14.79 (6.62)	12.69 (5.61)	12.79 (6.62)

^a^ LBP1 = US$0.0007.

In terms of work‐related variables, nurses who were working rotated shift had a significant lower quality of life for the social relationship domain than nurses who worked morning shift, at (mean = 13.65, *SD* 2.90) versus (mean = 17.14, *SD* 5.94), (*t* = 30.14, *p* < .01). However, there were no significant differences in all domains of quality of life according to marital status and monthly income.

In terms of occupational stress, nurses with BCs degree had higher total scores of NSS than nurses with diploma (mean = 71.13, *SD* 4.95) versus (mean = 65.67, *SD* 5.50), (*t* = 18.66, *p* < .01). In addition, nurses who were working morning shift had less occupational stress than other nurses who were working rotating shift (mean = 66.67, *SD* 7.07) versus (mean = 73.98, *SD* = 3.98), (*t* = 24.21, *p* < .01) (Table 2).

Correlation coefficients were conducted to evaluate relationships of occupation stress and quality of life with continuous variable (age, years of experience). The physical domain of the WHO‐BREF was significantly and negatively associated with age and years of experience (*p* < .05). Conversely, the social domain of the WHO‐BREF was significantly and negatively associated with only years of experience. Furthermore, a negative significant association was noted between occupational stress and age and years of experience (Table 3).

**Table 3:** Correlation of occupational stress and quality of life with age and years of experience in older people care settings

Variable	NSS	WHOQOL‐BREF			
	Occupational stress	Physical domain	Psychological domain	Social domain	Environmental domain
Age	−0.29**	−0.51**	0.03	−0.16	0.05
Years of experience	−0.32**	−0.20*	0.19	−0.26*	0.09

* *p* < .05.

** *p* < .01.

### 3.4 Relationship between stress and quality of life controlling for sample characteristics

Hierarchical regression analysis was performed to investigate the relative and overall contribution of demographic characteristics, work‐related variables and occupational stress on nurses' quality of life. The variable that was not significantly associated with quality of life was excluded from this analysis.

The relationship between stress and the physical domain of WHO‐BREF controlling for sample characteristics is presented in Table 4. The demographic and work‐related characteristics were inserted in the first model which was significant, *F* (2, 116) = 12.43, *p* < .001, *R* ^2^ = .37. However, in this model, only the age was significantly correlated with the physical domain of WHO‐BREF controlling for other sample characteristics (*p* < .001), indicating that older nurses had a significant lower quality of life than younger nurses for physical domain of WHO‐BREF. The occupational stress was inserted in the next model which also was significant, *F* (3, 115) = 67.9, *p* < .001, *R* ^2^ = .82. Occupational stress explained 42% additional variance above and beyond the 38% accounted for demographic and work‐related characteristics. This result shows that occupational stress is correlated with low levels on physical domain of WHO‐BREF, with a large effect size, *R* ^2^ change = .42.

**Table 4:** The relationship between occupational stress and physical domain of WHO‐BREF controlling for demographic and work‐related variables

	B	Βeta	*t*	*p*‐Value
(Constant)	69.73		4.70	.000
Age	−25.91	−0.51	−4.55	.000
Years of experience	10.20	−0.23	−1.89	.065
(Constant)	160.19		14.59	.000
Age	−9.93	−0.19	−3.01	.004
Years of experience	−3.78	−0.08	−1.43	.157
Occupational stress (NSS)	−0.27	−0.77	−11.75	.000

Note

1st model *F* (2, 116) = 12.43, *p* < .001, *R* ^2^ = .37.

2nd model *F* (3, 115) = 67.9, *p* < .001, *R* ^2^ = .82, *R* ^2^ change = .42.

The relationship between occupational stress and the psychological domain of WHO‐BREF controlling for sample characteristics is presented in Table 5. The demographic and work‐related characteristics were inserted in the first model which was significant, *F* (2, 116) = 6.04, *p* < .001, *R* ^2^ = .18. The education level was the only variable correlated with the psychological domain of WHO‐BREF (*p* < .001), representing that the higher education level of nurses had significantly higher scores on the psychological domain of WHO‐BREF. Occupational stress was inserted in the next model, which was significant, *F* (3, 115) = 36.18, *p* < .001, *R* ^2^ = .63. The occupational stress explained 43% additional variance above and beyond the 18% accounted for demographic and work‐related characteristics. This result shows that occupational stress is correlated with low levels on the psychological domain of WHO‐BREF, with a large effect size, *R* ^2^ change = .43.

**Table 5:** The relationship between occupational stress and psychological domain of WHO‐BREF controlling for demographic and work‐related variables

	B	Βeta	*t*	*p*‐Value
(Constant)	29.13		2.85	.000**
Education level	−16.02	−0.30	−4.24	.000**
Shift worked	6.20	0.08	0.99	.321
(Constant)	176.21		11.13	.000**
Education level	−3.99	−0.09	−2.25	.055
Shift worked	−4.59	−0.06	−1.09	.279
Occupational stress (NSS)	−0.37	−0.74	−12.29	.000**

Note

1st model *F* (2, 116) = 6.04, *p* < .001, *R* ^2^ = .18.

2nd model *F* (3, 115) = 36.18, *p* < .001, *R* ^2^ = .63, *R* ^2^ change = .43

** *p* < .001

The relationship between occupational stress and the social relationship domain of WHO‐BREF controlling for sample characteristics is presented in Table 6. The demographic and work‐related characteristics were inserted in the first model which was significant, *F* (2, 116) = 6.05, *p* < .001, *R* ^2^ = .18. However, in this model, only the shift worked was significantly correlated with the social domain of WHO‐BREF controlling for other sample characteristics (*p* = .001), indicating that nurse who was working evening/rotating shifts had a significant lower quality of life for the social domain of WHO‐BREF compared with nurses who was working morning shifts. Occupational stress was inserted in the next model, which was significant, *F* (3, 115) = 36.18, *p* < .001, *R* ^2^ = .53. Occupational stress explained 34% additional variance above and beyond the 18% accounted for demographic and work‐related characteristics. This result shows that occupational stress is correlated with low levels on the social domain of WHO‐BREF, with a large effect size, *R* ^2^ change = .34.

**Table 6:** The relationship between occupational stress and social relationship domain of WHO‐BREF controlling for demographic and work‐related variables

	B	Βeta	*t*	*p*‐Value
(Constant)	29.13		2.80	.000**
Shift worked	−15.03	−0.29	−3.45	.000**
Years of experience	−6.20	−0.08	−0.98	.346
(Constant)	150.38		9.50	.000**
Shift worked	−3.99	−0.09	−2.25	.002
Years of experience	−4.59	−0.06	−1.09	.290
Occupational stress (NSS)	−0.37	−0.74	−12.29	.000**

Note

1st model *F* (2, 116) = 6.05, *p* < .001, *R* ^2^ = .19.

2nd model *F* (3, 115) = 36.18, *p* < .001, *R* ^2^ = .54, *R* ^2^ change = .35.

** *p* < .001.

The relationship between stress and the environmental domain of WHO‐BREF is presented in Table 7. No demographic and work‐related characteristics were significantly associated with environmental domain; thus, all these variables were excluded from the analysis. The finding indicates that occupational stress is correlated with low levels on the environmental domain of WHO‐BREF, with *R* ^2^ = .37.

**Table 7:** The relationship between occupational stress and environmental domain of WHO‐BREF

	B	Βeta	*t*	*p*‐Value
Occupational stress (NSS)	−25.91	−0.51	−4.55	.000**

Note

*F* (1, 116) = 12.43, *p* < .001, *R* ^2^ = .38.

** *p* < .001.

## 4 DISCUSSION

To our knowledge, no study has been conducted on quality of life among nurses caring for older patients and the impact of their demographic and work‐related variables and occupational stress on their health. Thus, the purposes of this study were to identify the sources of occupational stressors and the level of quality of life among nurses caring for older patients in Lebanon and to examine the underlying factors to predict nurses' quality of life.

This study has shown that nurses reported the highest frequency of occupational stressors due to their workload. Looking at individual items on the workload subscale, “not enough time to complete all my nursing tasks” was the most frequently selected items. This is consistent with previous studies that found that “the lack of time to provide the care needed by patients” was one of the most common stressors among nurses working with older people (El‐Hneiti et al., 2019; Saarnio, Sarvimäki, Laukkala, & Isola, 2012). This stressor could be clarified by the physical and psychological needs among older people. Previous studies showed that 5%–55% of the older persons have neurological deficits such as vision and hearing problems and up to 20% of older adults have psychiatric disorders (Boulton‐Lewis, Aird, & Buys, 2016; Hofman et al., 2013). Being unable to provide the care for these needs among older people is a source of stress (Zwijsen et al., 2014). Furthermore, exposure to a long period of workload can easily cause many negative impacts on both patient and nurse, including lower quality of patient care and safety, as well as higher rates of burnout and turnover of nurses (Chiang, Hsiao, & Lee, 2017; Cuadros, Padilha, Toffoletto, Carlos, & Canales, 2017).

Moreover, this study has found that nurses reported death and dying as the second most frequency stressors of their work. This is in line with previous studies conducted in different countries and revealed that the death and dying situations were the most common source of occupational stress (Galdikien et al., 2014; Sarafis, 2016). Therefore, nurse educators should support nurses by developing a continuous education program about nursing care practice with older people that might help nurses to deal with death and dying.

Although the topics related to death and dying integrated into undergraduate nursing curriculum in Lebanon (Daher et al., 2013), nurses reported these topics as the second most frequency stressors of their working with older people. This finding suggests that there are knowledge gaps and inadequate preparation of nursing students to deal with death and dying situations. Therefore, nursing educators should give much attention to finding teaching methods that are appropriate to this challenging topic and that more effectively prepare nursing students for what they might encounter.

It is important to highlight that the mean score across all the stressors on NSS was higher than previous studies (Faremia, Olatubi, Adeniyi, & Salau, 2019; Wareth & Eltaybani, 2019). This indicates that nurses in this study were more stressed than nurses in other settings. Further studies are needed to examine the sources of occupational stressors among nurses working in older people care settings. To explore these stressors in depth, a qualitative research design is recommended in further research.

In terms of nurses' quality of life, current study revealed that quality of life was lower in older nurses who were working rotated shift and those having higher years of experience, which is in line with previous studies that found a significant association between quality of life and age (Albuquerque et al., 2019) and years of experience (Tessy, 2014). Thus, leaders should be conscious to work‐related factors that lead to reduce quality of life which include worked shift and years of experience. Further employing agencies and nursing profession should pay attention to older nurses working with older people because it is shown that they had lower quality of life than younger nurses.

It was worthwhile to note that there were some differences in predictive factors for physical, psychological and social relationships domains of quality of life among nurses working with older patients. This implied that physical health domain was affected more by age than psychological and social relationships domains. The psychological health domain was related more closely to educational level than physical and social relationships domains. The social relationships domain was affected more by shift worked than psychological and physical health domains.

This study found a significant relationship between occupational stress and quality of life controlling for the demographic and work‐related variables, with higher levels of stress associated with poor quality of life among nurses caring for older patients. This is consistent with previous studies that found that higher levels of occupational stress associated with poor quality of life among psychiatric nurses (Hamaideh, 2012; Itzhaki, 2018) and general nurses (Sarafis et al., 2016).

One of the strengths of this study was controlling sample characteristics, which was useful to identify how much occupational stress has a unique contribution to nurses' quality of life. Occupational stress management was shown to be the key to enhance quality of life in nurses working in older people care settings. Providing stress management training (SMT) such as cognitive behavioural skills training and relaxation techniques is highly recommended for nurses to deal with difficult work situations and raise their abilities regarding cognitive self‐control (Alkhawaldeh, Soh, et al., 2019; Krölla, Doeblerb, & Nüescha, 2017; Singh, 2017). Therefore, nurse educators and leaders should support nurses working with older people by conducting SMT to reduce the impact of the occupational stress on their quality of life. Further research to assess the effectiveness of this training is needed.

In terms of the limitations in this study, the sample is selected using convenience sampling technique that limits the potential to generalize the findings. Although generalizability is not as probable from this study's findings as it would be with a more substantial random sample, the sample of convenience did permit the use of a target and accessible research population (Cokley & Awad, 2013). Another limitation of the study is used self‐reporting method, as this approach was appropriate for meeting the study purposes. Self‐report measures could be associated with social desirability bias, where participants may guess what the researcher is looking for and change their answers accordingly. However, the instruments used in this study may have been less susceptible to this bias due to their nature, as most responses to the questions are considered socially acceptable. Despite these limitations, this study provided valuable data that could be applied to enhance quality of life among nurses caring for older people in the future.

### 4.1 Implications for clinical practice

- Employment of more nurses especially in older people care settings may help to decrease the impact of workload stressor on nurses' quality of life.
- Leaders should be aware to work‐related factors that lead to reduce quality of life which include worked shift and years of clinical experience.
- Employing agencies and nursing profession should pay attention to older nurses working with older people because it is shown that they had lower quality of life than younger nurses.

## 5 CONCLUSION

Nurses caring for older people face with a variety of sources of occupational stressors. Finding of this study found that workload and dealing with death and dying are the most common sources of nurses' work that resulted in highest frequency of occupational stressors. Thus, it is vital for nursing profession to consider these occupational stressors in the conditions of working in older people care settings.

While investigating the predictors of quality of life among nurses caring for older patients, occupational stress remained a significant predictor of nurses’ quality of life after controlling for demographic and work‐related variables, suggesting that the relationship between occupational stress and nurses' quality of life was independent of these potential confounding variables. Therefore, nurse educators and leaders should support nurses working with older people by conducting stress management training to reduce the impact of the stress on their quality of life.

## CONFLICT OF INTEREST

The authors declare that they have no conflicts of interest.
